# GMMA and Glycoconjugate Approaches Compared in Mice for the Development of a Vaccine against *Shigella flexneri* Serotype 6

**DOI:** 10.3390/vaccines8020160

**Published:** 2020-04-03

**Authors:** Maria Michelina Raso, Gianmarco Gasperini, Renzo Alfini, Fabiola Schiavo, Maria Grazia Aruta, Martina Carducci, Maria Concetta Forgione, Silvia Martini, Paola Cescutti, Francesca Necchi, Francesca Micoli

**Affiliations:** 1 GSK Vaccines Institute for Global Health (GVGH) S.r.l., via Fiorentina 1, 53100 Siena, Italy; maria-michelina.m.raso@gsk.com (M.M.R.); gianmarco.x.gasperini@gsk.com (G.G.); renzo.x.alfini@gsk.com (R.A.); fabiola.schiavo@yahoo.it (F.S.); maria-grazia.x.aruta@gsk.com (M.G.A.); martina.x.carducci@gsk.com (M.C.); francesca.x.necchi@gsk.com (F.N.); 2 Department of Life Science, University of Trieste, Building C11, via L. Giorgieri 1, 34127 Trieste, Italy; pcescutti@units.it; 3 GSK, via Fiorentina 1, 53100 Siena, Italy; mariaconcetta.x.forgione@gsk.com (M.C.F.); silvia.x.martini@gsk.com (S.M.)

**Keywords:** *Shigella*, glycoconjugate, GMMA, O-antigen, vaccine

## Abstract

*Shigella* infections are one of the top causes of diarrhea throughout the world, with *Shigella flexneri* being predominant in developing countries. Currently, no vaccines are widely available and increasing levels of multidrug-resistance make *Shigella* a high priority for vaccine development. The serotype-specific O-antigen moiety of *Shigella* lipopolysaccharide has been recognized as a key target for protective immunity, and many O-antigen based candidate vaccines are in development. Recently, the Generalized Modules for Membrane Antigens (GMMA) technology has been proposed as an alternative approach to traditional glycoconjugate vaccines for O-antigen delivery. Here, these two technologies are compared for a vaccine against *S. flexneri* serotype 6. Genetic strategies for GMMA production, conjugation approaches for linkage of the O-antigen to CRM_197_ carrier protein, and a large panel of analytical methods for full vaccine characterization have been put in place. In a head-to-head immunogenicity study in mice, GMMA induced higher anti-O-antigen IgG than glycoconjugate administered without Alhydrogel. When formulated on Alhydrogel, GMMA and glycoconjugate elicited similar levels of persistent anti-O-antigen IgG with bactericidal activity. Glycoconjugates are a well-established bacterial vaccine approach, but can be costly, particularly when multicomponent preparations are required. With similar immunogenicity and a simpler manufacturing process, GMMA are a promising strategy for the development of a vaccine against *Shigella*.

## 1. Introduction

*Shigella* infections are one of the top causes of moderate to severe diarrhea (MSD) throughout the world. Shigellosis, or bacillary dysentery, is an acute human inflammatory disease of the large intestine, characterized by watery diarrhea, fever, abdominal pain, and bloody and mucus stools [1]. The Global Burden of Disease Study 2016 estimates approximately 112 million cases with 269,191 total deaths per year, of which 30% are children younger than 5 years, and 98.5% are in low- and middle-income countries (LMIC) [2]. There are four different *Shigella* species: *S. boydii*, *S. dysenteriae*, *S. flexneri*, and *S. sonnei*. The first three species are typed into 50 different serotypes or subserotypes based on antigenic variation of their O-antigen (OAg) [3]. Incident data from specific sites of the Global Enteric Multicentre Study (GEMS) in sub-Saharan Africa and South Asia shows that 24% of cases are caused by *S. sonnei* and 66% by *S. flexneri*, mostly by serotypes 1b, 2a, 3a, and 6 [4].

Increasing levels of multidrug-resistance [5,6] limit the effectiveness of antibiotics, making this disease a high priority for vaccine development [7,8]. Currently, no vaccines are widely available, but several candidates are being tested in different clinical phases, including subunit vaccines and killed or live-attenuated bacteria [9]. Studies in animal models and humans have demonstrated that protection by immunization is feasible. Serum and mucosal antibody responses to *Shigella* are predominantly directed against the serotype-specific *Shigella* OAg, and many *Shigella* vaccine candidates target the OAg [9]. 

Recently, the Generalized Modules for Membrane Antigens (GMMA) technology has been proposed for the development of a multi-component vaccine against *Shigella* [10]. GMMA are outer membrane exosomes naturally released from genetically engineered Gram-negative bacteria, where the OAg is displayed in its natural outer membrane context. Bacteria are mutated in order to increase exosome formation, through the deletion of the *tolR* gene, and to reduce potential reactogenicity, usually through modifying the lipid A structure by deletion of the *htrB*, *msbB* or *pagP* genes [11,12]. An *S. sonnei* GMMA-based vaccine (1790GAHB) [10] has been tested in clinical studies, showing high immunogenicity, memory response, and good tolerability [13,14,15]. 

A more traditional technology for the development of OAg-based vaccines is the glycoconjugation approach. The covalent linkage of a polysaccharide (PS) to an appropriate carrier protein converts the PS from a T-independent to a T-dependent antigen, able to induce immunological memory and make the vaccine also effective in infants [16,17]. Different parameters can influence the immune response of glycoconjugate vaccines, in particular, the PS length and the degree of carbohydrate loading (density), the nature of the carrier protein, and the conjugation chemistry used [18,19]. In addition, non-carbohydrate modifications of the OAg like O-acetyl groups [20] are important aspects to investigate for the design of an optimal PS-based vaccine [21]. 

Here, the GMMA technology has been compared to the more traditional glycoconjugate approach for the development of a vaccine candidate against *S. flexneri* serotype 6. Bacteria have been genetically manipulated for the production of GMMA, conjugation approaches have been developed for the OAg linkage to the CRM_197_ carrier protein (genetically inactivated toxoid of diphtheria toxin) [22], and appropriate analytical methods for vaccines characterization have been put in place. *S. flexneri* 6 OAg is characterized by a linear polysaccharide backbone →2)-α-L-Rha*p*^III^-(1→2)-α-L-Rha*p*^II^-(1→4)-β-D-Gal*p*A-(1→3)-β-D-Gal*p*NAc-(1→, with Rha^III^ variably O-acetylated in position 3 or 4 [23]. The OAg biosynthesis depends on the Wzx/Wzy pathway and involves the first step of sugar polymerization during which the OAg chain length is regulated by the Wzz proteins, responsible for unique polysaccharide modal lengths. The OAg repeating units can also be polymerized into a group 4 capsule (G4C) because of the presence, in addition to the *wzx-wzy* cluster, of a G4C operon [24]. 

The two approaches for vaccine production have been compared in mice for their ability to elicit specific anti-OAg antibodies, functionality, T-dependent nature, quality, and longevity of the induced immune response. The impact of the OAg length on the induced immune response by both vaccine technologies has also been evaluated, together with the role of OAg O-acetylation and conjugation chemistry, in the specific case of glycoconjugates, supporting the design of an optimal vaccine against *Shigella*.

## 2. Materials and Methods 

### 2.1. Bacterial Strains, Mutant Generation and Growth Condition

*Shigella flexneri* 6 wild type strains were obtained from the Wellcome Trust Sanger Institute and Public Health England (Table 1) [25]. Strain Sf6_Sh10.8537 was selected for the generation of deletion mutants. To generate the mutants, the kanamycin resistance gene *aph* was used to replace the *tolR* gene, the *ept-etk* genes, and the *wzzB* gene (Table 1). The resistance cassette replacement constructs were amplified from the pKD4 vector using forward and reverse primers composed of 50 bp homologous to the flanking regions of the gene to be deleted and approximately 20 bp (reported in bold in Table 2) at the 3′ end matching the flanking region of the resistance gene. Primer sequences are listed in Table 2. PCR products were purified and were used to transform recombination-prone *S. flexneri* 6 recipient cells carrying pKD46 by following methods described previously [26]. After each gene deletion, the kanamycin resistance gene was removed through FLP-mediated recombination using the pCP20 plasmid to yield markerless mutant strains.

All bacterial strains were grown at 30 °C in liquid Luria–Bertani (LB) medium in a rotary shaker for 16 hours. For outer membrane vesicles (OMVs) and GMMA production, overnight cultures were diluted in HTMC medium (15 g/L glycerol, 30 g/L yeast extract, 0.5 g/L MgSO_4_, 5 g/L KH_2_PO_4_, 20 g/L K_2_HPO_4_) to an optical density at 600 nm (OD600) of 0.3 and grown at 30 °C in a rotary shaker for 8 hours using baffled flasks with a liquid to air volume ratio of 1:5.

### 2.2. OMV/GMMA Production

OMVs were produced from all the *S. flexneri* 6 wild type strains available, while GMMA were produced from Sf6_Sh10.8537 ∆*tolR* strain and derivatives. After growth, bacteria were pelleted through centrifugation at 5000*× g* for 45 minutes. Cell-free supernatants were recovered and filtered through 0.22 μm Stericup filters (Millipore, Burlington, MA, USA). After ultracentrifugation of filtered supernatants at 175,000× *g* for 2 hours at 4 °C, the resulting pellet, containing OMVs or GMMA, was washed with phosphate-buffered saline (PBS), further ultra-centrifuged at 175,000× *g* for 2 hours and finally resuspended in PBS.

### 2.3. GMMA Characterization

GMMA size was determined by dynamic light scattering (DLS) and high performance liquid chromatography–size exclusion chromatography/multiangle light scattering (HPLC–SEC/MALS) as previously described [27]. GMMA purity was assessed by HPLC–SEC analysis [28]; total protein content was estimated by micro BCA using bovine serum albumin (BSA) as a reference following the manufacturer’s instructions (Thermo Scientific, Waltham, MA, USA); OAg sugar content was quantified by determination of methyl pentoses (6-deoxyhexoses) with Dische colorimetric method [29]. The amount of lipid A molecules in GMMA was assumed equal to lipopolysaccharide (LPS) core reducing end 2-keto-3-deoxy-octonate (KDO) and quantified by semicarbazide/HPLC–SEC method after sugar extraction [30].

### 2.4. OAg Purification and Characterization

OAg extraction and purification from wild type bacteria, OMVs, or GMMA was performed as previously described [28,31]. Gel filtration chromatography was used to fractionate the OAg populations of different average sizes obtained through extraction from Sf6_Sh10.8537 ∆*tolR* GMMA. The sample was run on a HiPrep 16/60 Sephacryl S300 HR column (600 × 16 mm; GE Healthcare, Marlborough, MA, USA), followed by a HiPrep 16/60 Sephacryl S100 HR column (600 × 16 mm; GE Healthcare, Marlborough, MA, USA). The mobile phase was PBS at a flow rate of 0.5 mL/min. 

OAg populations were characterized by HPLC–SEC with differential refractive index (dRI) detection to estimate the molecular size distribution. The OAg samples were run on a TSK gel G3000 PWXL column (30 cm × 7.8 mm; particle size 7 µm; cod. 808021) with TSK gel PWXL guard column (4.0 cm × 6.0 mm; particle size 12 µm; cod.808033; Tosoh Bioscience Tokyo, Japan). The mobile phase was 0.1 M NaCl, 0.1 M NaH_2_PO_4_, 5% CH_3_CN, pH 7.2 at the flow rate of 0.5 mL/min (isocratic method for 35 min). The OAg peak molecular weight (MP) was calculated using dextrans as standards in the range 12–150 kDa. Sugar content was estimated by Dishe colorimetric assay [29]. Nuclear magnetic resonance (NMR) spectroscopy was used to confirm OAg identity and purity and to calculate O-acetylation degree [23]. All NMR experiments were performed with a AEON AVANCE III 600 MHz spectrometer (Bruker, Billerica, MA, USA) equipped with a high-precision temperature controller using a 5 mm QCI CryoProbe. To confirm the presence of saccharide populations with different molecular mass (MM), a diffusion filter pulse sequence was applied (diffusion measurement with stimulated echo and LED using bipolar gradient pulses for diffusion, ledbpgp2s1d, Bruker). Pulse sequence-specific acquisition parameters were chosen according to the extent of the desired MM separation. In particular, the diffusion time (100, 200, and 300 ms), the gradient pulse length (2500 µs), and the z-gradient intensity (gpz6 = 80%) were changed accordingly. Spectra were weighted with 0.8 Hz line broadening and Fourier-transformed. *S. flexneri* 6 ^1^H and ^13^C resonances were assigned by ^1^H, 2D-COSY, 2D-TOCSY, 2D-NOESY, 2D-ROESY, and 2D-HSQC experiments. Both 1D and 2D-NMR spectra were recorded at 50.0 ± 0.1 °C. The transmitter was set at the water frequency (4.70 ppm). Proton spectra were acquired using a 90-degree pulse duration automatically calculated and collected with 32K data points over a 12 ppm spectral width, accumulating 128 number of scans. Spectra were processed by applying an exponential function to the FID with a line broadening of 0.80 Hz to increase the signal-to-noise ratio and then Fourier transformed. The 2D-COSY spectra, with presaturation during relaxation delay, were acquired with data sets of 4096 × 256 points; phase sensitive 2D-TOCSY spectra were performed with a spinlock time of 100 ms and data sets of 2048 × 256 points. Phase-sensitive 2D-ROESY spectra were acquired with data sets of 4096 × 256 points. Mixing times of 180 and 150 ms were used. HSQC was acquired with 1024 × 128 points. Data acquisition and processing were performed with a TopSpin 3.5 software package (Bruker BioSpin).

### 2.5. Glycoconjugates Synthesis and Characterization

OAg populations of different average sizes were conjugated to CRM_197_ (kindly provided by GSK Vaccines) using both a selective and a random approach. For selective conjugation, OAg was derivatized with adipic acid dihydrazide (ADH) by reductive amination of the KDO terminal sugar and linked to the amino groups on the protein after attachment of a second linker, adipic acid bis (*N*-hydrosuccinimide) (SIDEA), to ADH [32]. The OAg-ADH intermediates were desalted by a PD10 desalting column prepacked with a Sephadex G-25 Superfine (GE Healthcare, Marlborough, MA, USA) or HiPrep xK 16/14 desalting column 20 mL, prepacked with Sephacryl G-10 Superfine (GE Healthcare, Marlborough, MA, USA) based on OAg average size. OAg-ADH-SIDEA reaction mixtures were diluted 1:1 *v/v* with water and desalted on a PD10 desalting column prepacked with Sephadex G-25 Superfine or with Sephacryl G-10 Superfine to remove residual free SIDEA. Conjugation mixtures were purified by HiPrep 16/60 Sephacryl S300 HR column or HiPrep 16/60 Sephacryl S100 HR column, according to OAg size. 

For random chemistry, sugar chains were randomly oxidized with sodium periodate (NaIO_4_), and the resulting aldehyde groups were conjugated to lysine residues on the protein by reductive amination, as previously described for *Salmonella* OAg [32]. Conjugate purification was performed by Sephacryl HR 16/60 S300 column (600 × 16 mm; GE Healthcare, Marlborough, MA, USA). 

Intermediates of conjugation were characterized as previously described [32]. Purified conjugates were characterized by the Dische colorimetric assay for sugar quantification [29] and the micro BCA, using BSA as a reference following the manufacturer’s instructions (Thermo Scientific, Waltham, MA, USA), for total protein content; the ratio of saccharide to protein was then calculated. HPLC–SEC was used to verify conjugates formation and detect the presence of unreacted protein [32]. Free saccharide was estimated by HPLC–SEC analysis using dRI detection in comparison to a standard curve of the corresponding unconjugated OAg or by DOC precipitation followed by Dische colorimetric assay [33]. 

### 2.6. Immunogenicity Studies in Mice

*S. flexneri* 6 GMMA and glycoconjugates were tested in mice. Animal studies were performed at Toscana Life Science Animal Care Facility under the animal project 479/2017-PR 09/06/2017, approved by the Italian Ministry of Health. Five-week-old female wild-type or nude CD1 (T-cell deficient) mice were immunized subcutaneously with 200 μL of vaccine at days 0 and 28. Sera were collected at days -1, 27, and 42. In certain studies, sera were also collected at day 98 to investigate the longevity of the induced immune response. Eight mice per group were injected with either formulations of GMMA or conjugates, with or without Alhydrogel (Aluminium hydroxide at 0.7 mg/mL Al^3+^ for GMMA and 2 mg/mL Al^3+^ for OAg-CRM_197_). Complete adsorption of GMMA or conjugates on Alhydrogel was confirmed by SDS-PAGE and silver staining analysis of supernatants from the different formulations. Different OAg doses were tested. 

Individual mouse sera were tested for anti-OAg total IgG by enzyme-linked immunosorbent assay (ELISA), as previously described [21]. *S. flexneri* 6 G4C, at a concentration of 5 μg/mL in carbonate buffer pH 9.6, was used as a coating antigen. OAg specific serum IgG subclasses (IgG1, IgG2a, IgG2b, IgG3) and IgM were also determined with a similar methodology.

Single sera were tested against a wild-type *S. flexneri* 6 strain in serum bactericidal assay (SBA) based on luminescent readout [34]. *S. flexneri* 6 H130920152 (Table 1) bacteria working cell bank, stored frozen at −80 °C in 20% glycerol stock, was grown overnight (16–18 hours) at 37 °C in LB medium, with stirring at 180 rpm. The overnight bacterial suspension was then diluted in fresh LB medium to OD_600nm_ of 0.05 and incubated at 37 °C with 180 rpm agitation in an orbital shaker until it reached an OD_600_ of 0.2 +/− 0.02. SBA was performed in 96-well round-bottom sterile plates (Corning, New York, NY, USA) by incubating serial dilutions in PBS of heat-inactivated (HI) test sera in the presence of exogenous baby rabbit complement (BRC) and bacteria for 3 hours at 37 °C. Log-phase cultures prepared as described above were diluted in PBS and added in the reaction well to an approximate concentration of 1 × 10^5^ colony forming unit (CFU)/mL. BRC at the final concentration of 25% was present in the reaction mixtures. For each serum dilution curve, a control well with no HI serum was added. At the end of the incubation, the SBA plate was centrifuged at RT for 10 min at 4000× g. The supernatant was discarded to remove ATP derived from dead bacteria and SBA reagents; the remaining live bacterial pellets were resuspended in PBS, transferred in a white round-bottom 96-well plate (Greiner, Kremsmünster, Austria) and mixed 1:1 *v:v* with BacTiter-Glo Reagent (Promega, Madison, MA, USA). The reaction was incubated for 5 min at RT on an orbital shaker, and the luminescence signal measured by a luminometer (Viktor, Perkin Elmer, Waltham, MA, USA). A 4-parameter non-linear regression was applied to raw luminescence data obtained for all sera dilutions tested; an arbitrary serum dilution of 10^15^ was assigned to the control well containing no sera. Fitting was performed by weighting the data for the inverse of luminescence^2. 

Results of the assay were expressed as the IC50, the reciprocal serum dilution that resulted in a 50% reduction of luminescence and thus corresponding to 50% growth inhibition of the bacteria present in the assay. GraphPad Prism 7 software was used for curve fitting and IC50 determination. Titers below the minimum measurable signal were assigned a titer of 50, corresponding to half of the first dilution of sera tested. 

### 2.7. Statistical Analysis 

Analysis was performed using GraphPad Prism 7. The Mann–Whitney *U*-test was used to compare two groups and a Kruskal–Wallis analysis with post-hoc Dunn’s test to compare multiple groups. The Wilcoxon matched-pairs signed rank two-tailed test was used to compare results from the same group at different time points.

## 3. Results

### 3.1. S. flexneri 6 OAg Characterization

**Figure 1:**
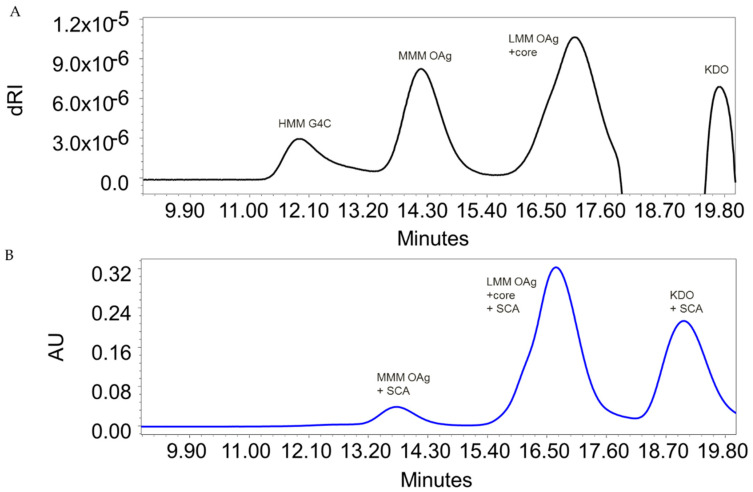
HPLC–SEC profiles of sugar extracted from *S. flexneri* 6 Δ*tolR* GMMA: (**A**) detection by refractive index and (**B**) detection at 252 nm after sugar derivatization with semicarbazide (SCA). Similar profiles were obtained for sugar extracted from all the wild-type strains analyzed (Table 1).

OAg was extracted from five different *S. flexneri* 6 strains, isolated in different countries (Table 1), by acetic acid hydrolysis performed directly on bacterial cultures [31]. Three main sugar populations of different average molecular mass (MM) were detected by HPLC-SEC dRI analysis (Figure 1A), indicated as high MM (HMM), medium MM (MMM), and low MM (LMM). Differently from the other two populations, the peak at HMM did not react with semicarbazide [31], even when treated at very high concentrations, indicating the lack of terminal KDO (Figure 1B). ^1^H NMR of the extracted sugar confirmed the expected structure, as reported in the literature for *S. flexneri* 6 OAg [23]. Diffusion-ordered spectroscopy (DOSY) NMR confirmed the presence of a population at HMM, where diagnostic peaks of the lipid A core region were absent, supporting the hypothesis that *S. flexneri* 6 strains produce a G4C lacking the LPS core region. For all *S. flexneri* 6 strains analyzed, G4C and OAg showed a similar O-acetylation pattern (Figure S1).

*S. flexneri* 6 bacteria naturally release OMV. For all 5 strains characterized, sugar populations were similar on bacteria and corresponding OMV, in terms of size and the relative amount of HMM G4C and MMM OAg (Table 3). However, spontaneous OMV release occurs at levels too low for vaccine manufacture. To increase the yield of bacterial exosomes, the wild-type strain Sf6_Sh10.8537 was engineered to obtain a hyper-blebbing phenotype (Table 1). The resulting GMMA displayed HMM G4C, MMM OAg, and LMM OAg (Figure 1), with a relative ratio of HMM G4C to MMM OAg of 20% similar to that on corresponding wild-type bacteria (Table 3). 

The three sugar populations at different average sizes were extracted from GMMA, isolated by S300 size exclusion chromatography, and characterized in more depth. Fractions collected at LMM revealed the presence of both very short OAg chains and lipid A core only, which were further isolated by S100 chromatography.

**Figure 2:**
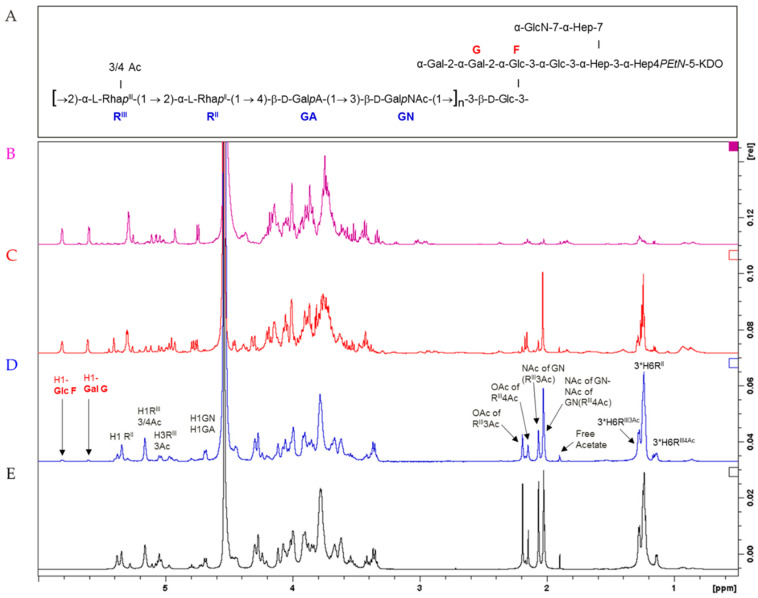
Structure of *S. flexneri* 6 OAg repeating unit and LPS core region (**A**); 1H NMR spectra of isolated core region (**B**); LMM OAg (**C**); MMM OAg (**D**; HMM G4C (**E**).

HMM G4C (average size of 174 kDa), MMM OAg (average size of 22 kDa), and LMM OAg (average size of 1.7 kDa) shared the same repeating unit structure, as confirmed by ^1^H NMR (Figure 2). MMM OAg was characterized by an average of 22 repeating units per chain, while LMM OAg had an average of 1.5 repeats, as calculated by comparing the Rha methyl signals at 1.16–1.31 ppm with the anomeric signals of Glc (5.84 ppm) and Gal (5.64 ppm) in the lipid A core. O-acetylation pattern was similar for HMM G4C and MMM OAg, with 48% O-acetylation on O-3 of Rha^III^ (both for MMM OAg and HMM G4C) and 15% or 18% on O-4 of Rha^III^ (for MMM OAg or HMM G4C, respectively). ^1^H NMR of LMM OAg also showed characteristic signals of the terminal Rha^III^ residue, present in four variants: non-O-acetylated and mono-acetylated at O-2, O-3, or O-4 (Figure 2) [35]. Total O-acetylation was calculated to be 52%. The signals were assigned based on the literature data [23,35] and 2D NMR experiments (data not shown).

### 3.2. Synthesis and Characterization of Glycoconjugates Differing in Sugar Length

**Figure 3:**
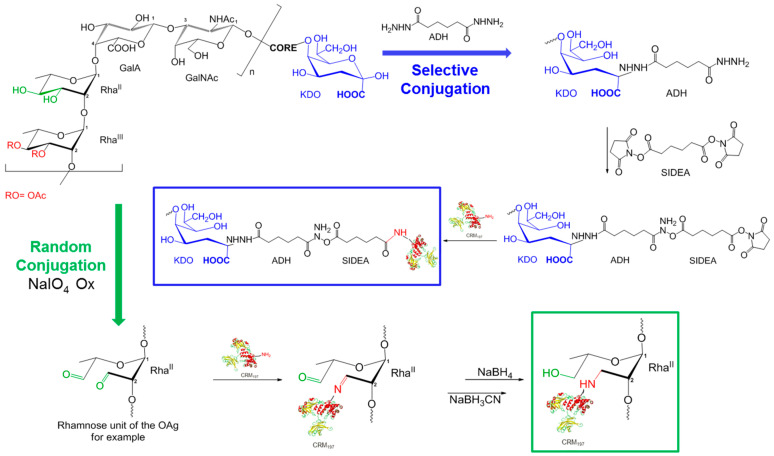
Conjugation schemes: random chemistry used with HMM G4C and MMM OAg, and selective chemistry used with MMM OAg and LMM OAg.

Isolated HMM G4C, MMM OAg, and LMM OAg were conjugated to CRM_197_ to investigate the possible impact of sugar length on the immune response induced by the corresponding glycoconjugates. A random approach, targeting multiple points along the PS chain, was used for the conjugation of the long G4C to CRM_197_. The aldehyde groups generated along the PS chain by random oxidation were used for direct reductive amination with lysine residues of the protein, resulting in a cross-linked and heterogeneous structure (Figure 3). A selective approach was used for the terminal linkage of LMM OAg to CRM_197_ so as not to alter important epitopes in the short OAg chains. The KDO sugar at the end of the OAg core region was used for introducing ADH and then SIDEA linkers, and binding to the carrier protein CRM_197_. Both chemistries were used to conjugate MMM OAg to CRM_197_ in order to verify the possible impact of conjugation chemistry on the immune response. 

To verify the role that O-acetyl groups can have on the immune response induced by *S. flexneri* 6 OAg, the HMM G4C was also de-O-acetylated in the presence of 200 mM sodium hydroxide before conjugation (Figure S2). Analysis by ^1^H-NMR confirmed the de-O-acetylation of HMM G4C. 

**Figure 4:**
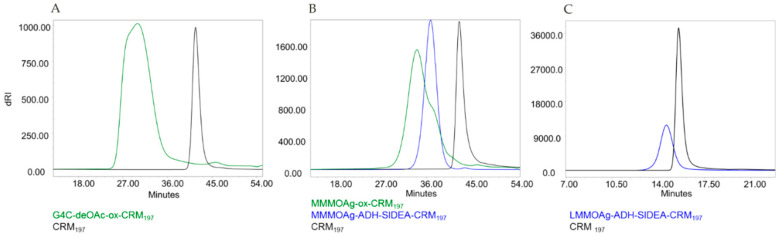
HPLC–SEC fluorescence emission profiles of G4C-ox-CRM_197_ (**A**), MMMOAg-ox-CRM_197_ and MMMOAg-ADH-SIDEA-CRM_197_ (**B**), and LMMOAg-ADH-SIDEA-CRM_197_ (**C**) compared to unconjugated CRM_197_.

All conjugates obtained showed no residual unconjugated protein in the reaction mixture, as verified by fluorescence emission profiles by HPLC–SEC analysis (Figure 4). Free saccharide was removed by purification through size exclusion chromatography (Table 4). The MM of the conjugates and the sugar to protein ratios were different according to sugar size and chemistry used (Figure 4, Table 4). Both selective conjugates were characterized by close to 5 OAg chains attached to CRM_197_. 

### 3.3. Immunogenicity of Glycoconjugates in Mice, Investigating Impact of Sugar Length, Conjugation Chemistry, and O-Acetylation on the Immune Response 

**Figure 5:**
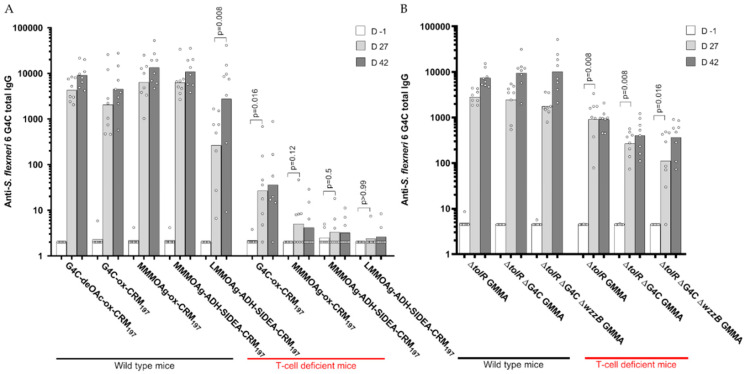
Immunogenicity of *S. flexneri* 6 glycoconjugates (**A**) and GMMA (**B**) differing in sugar length compared in mice. Eight wild-type and T-cell deficient mice per group were subcutaneously immunized at days 0 and 28, with 1 μg OAg dose on Alhydrogel (glycoconjugates) or 0.5 μg OAg dose without Alhydrogel (GMMA). Summary graphs of anti-OAg specific IgG geometric mean units (bars) and individual antibody levels (dots).

All OAg-CRM_197_ conjugates were compared in mice at 1 µg OAg dose formulated with Alhydrogel. Mice were immunized subcutaneously at 4-week intervals. Independent of the OAg chain length and the chemistry used, all the conjugates induced high levels of anti-OAg specific IgG. Four weeks after the first immunization, the LMM OAg conjugate induced a significantly lower IgG response compared to the other conjugates. The antibody response increased after re-injection (*p* = 0.008), reaching similar levels to those induced by the other conjugates. No booster after re-injection was observed for HMM G4C conjugate (*p* = 0.15) and MMM OAg conjugated by random chemistry (*p* = 0.08) (Figure 5A). Two weeks after the second immunization, the functionality of serum antibodies measured by SBA was similar for all the groups (Figure S3A). The same conjugates were also compared in T-cell deficient mice to evaluate their ability to induce a pure T-dependent response according to the different chain lengths. Only the HMM G4C-CRM_197_ conjugate induced an anti-OAg IgG response significantly different from background levels in T-cell deficient mice. This level was much lower compared to that obtained in wild-type mice (Figure 5A).

The de-O-acetylated G4C conjugate induced similar IgG and SBA titers to the corresponding native O-acetylated G4C conjugate (Figure 5A and Figure S3A). 

### 3.4. Genetic Engineering of Bacteria to Generate GMMA Expressing OAg of Different Lengths

**Figure 6:**
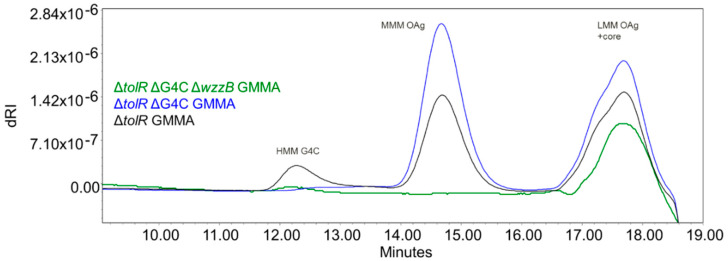
HPLC–SEC dRI profiles of sugar extracted from S. flexneri 6 ΔtolR (black), ΔtolR ΔG4C (blue), and ∆tolR ∆G4C ∆wzzB (green) GMMA.

With the aim to investigate the impact of sugar length on the immune response induced by *S. flexneri* 6 OAg, we also generated a set of *S. flexneri* 6 GMMA differing in OAg chain lengths. The GMMA-producing strain Sf6_Sh10.8537 Δ*tolR* was further mutated to abolish capsule formation by removing the *ept-etk* genes in the G4C operon. OAg chain length regulation was then prevented by removing the *wzzB* gene, resulting in the presence of OAg chains with only a few repeats (Table 1). The characterization of the resulting GMMA (Figure 6, Table 5) confirmed the production of GMMA with no HMM G4C (but carrying both MMM OAg and LMM OAg) and GMMA with LMM OAg only. The absence of G4C did not impact the overall sugar to protein ratio on GMMA, while the prevention of MMM OAg formation resulted in GMMA characterized by a lower sugar to protein ratio (Table 5).

All mutated GMMA had similar average particle size by HPLC–SEC MALLS, but not by DLS where the length of the sugar chains impacted the hydrodynamic diameter [27]. All GMMA were characterized by a similar number of lipid A molecules per GMMA protein (Table 5). 

### 3.5. Immunogenicity of GMMA in Mice, Investigating the Impact of Sugar Length on the Immune Response

GMMA differing in OAg length were tested in wild-type and T-cell deficient mice at a dose of 0.5 µg of OAg with no Alhydrogel. All GMMA induced similarly high anti-OAg specific IgG responses 27 days after one single dose with a booster after re-injection, independent of sugar length. Differently from what we observed with glycoconjugates, all GMMA induced a significant response in T-cell deficient mice, even if antibody responses were lower compared to those obtained in wild-type mice (Figure 5B). Similar results were obtained in terms of serum antibody functionality by SBA (Figure S3B).

**Figure 7:**
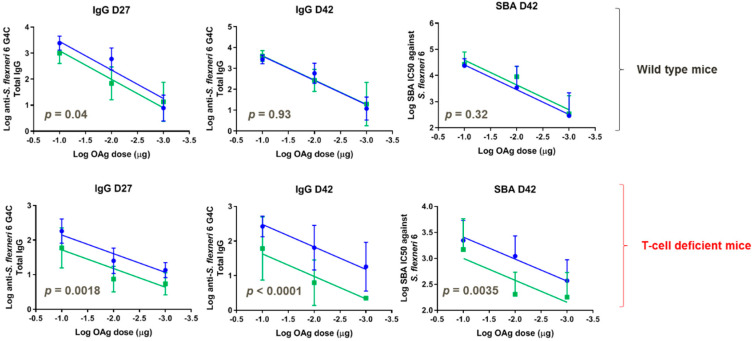
*S. flexneri* 6 GMMA with MMM OAg and LMM OAg (blue) or LMM OAg only (green) compared in mice. Eight wild-type and eight T-cell deficient mice per group were subcutaneously immunized at days 0 and 28, with different OAg doses without Alhydrogel. Each curve represents log-transformed doses on the abscissa and the log-transformed ELISA units or SBA titers on the ordinate. The parallelism of the lines was tested by comparison of the slopes, which resulted in not-significant difference. Subsequently, the *Y*-intercept of the curves obtained for the two GMMA were compared and the *p*-value is reported in each graph.

GMMA displaying MMM OAg and LMM OAg or LMM OAg were only further compared in wild-type and T-cell deficient mice in a dose-response study (0.1, 0.01, and 0.001 μg OAg doses), without Alhydrogel. For both GMMA, a significant correlation between OAg doses and antibody responses (either total IgG or SBA titers) was observed both in wild-type and T-cell deficient mice. In wild-type mice, four weeks after the first immunization, GMMA with LMM OAg chains induced lower anti-OAg total IgG compared to GMMA with MMM OAg and LMM OAg at all tested doses. The response increased following the second immunization to reach comparable IgG levels and SBA titers to those obtained with GMMA displaying both MMM OAg and LMM OAg (Figure 7). In T-cell deficient mice, GMMA with LMM OAg only induced significantly lower IgG compared to GMMA with MMM OAg and LMM OAg, both after the first and the second immunization. Similar results were obtained by testing antibody functionality by SBA at day 42.

### 3.6. Direct Comparison in Mice of GMMA and Glycoconjugate

**Figure 8:**
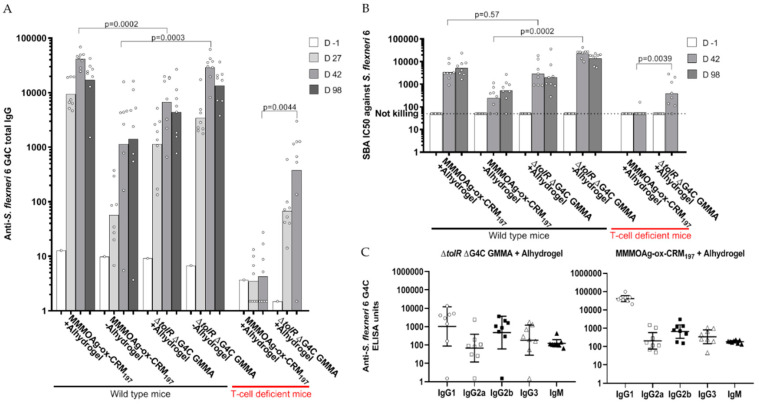
*S. flexneri* 6 glycoconjugate and GMMA displaying MMM OAg compared in mice. Eight mice per group were subcutaneously immunized at days 0 and 28, with 1 µg OAg dose with or without Alhydrogel. Anti-OAg specific IgG geometric mean units (bars) and individual antibody levels (dots) (**A**); SBA titers of single sera against *S. flexneri* serotype 6 strain (**B**); IgM and IgG subclasses analysis of single sera at day 42: individual antibody levels (dots) and geometric means (horizontal lines) (**C**).

Based on the results obtained from the previous studies in mice, GMMA and glycoconjugate presenting MMM OAg were selected for a head-to-head comparison. The constructs were tested at 1 µg OAg dose with or without Alhydrogel in outbred mice. Constructs with Alhydrogel were also tested in T-cell deficient mice. In the absence of Alhydrogel, GMMA induced significantly higher specific anti-OAg total IgG than the conjugate (*p* = 0.0003 at day 42, two weeks after second immunization). The behavior was the opposite when the constructs were adsorbed on Alhydrogel (*p* = 0.0002 at day 42; Figure 8A). The results were confirmed after testing the bactericidal activity of the induced antibodies: SBA titers of antibodies induced by GMMA were significantly higher than those induced by the conjugate in the absence of Alhydrogel (*p* = 0.0002 at day 42), but they were not different in the presence of Alhydrogel (Figure 8B). Similar results were obtained by testing GMMA and conjugated at 0.1 µg OAg dose (data not shown).

Analysis of anti-antigen specific IgG subclasses and IgM was performed on sera collected at day 42. Both GMMA and the glycoconjugate induced not only IgG1 but also IgG2a, IgG2b, IgG3, and IgM (Figure 8C). Immunization of T-cell deficient mice confirmed the ability of GMMA, but not of the glycoconjugate, to induce a significant response, both in terms of anti-OAg total IgG and SBA titers (Figure 8). However, both GMMA and the glycoconjugate were able to induce persistent responses in mice, as verified by the IgG and SBA titers at day 98, 70 days after the second immunization (Figure 8).

## 4. Discussion

In this study, we compared GMMA and glycoconjugate technologies for the development of a vaccine candidate against *S. flexneri* serotype 6, a main cause of shigellosis in LMIC. The conjugation approach has been largely explored for the development of OAg-based vaccines [16,36,37,38]. More recently, GMMA have been proposed as an alternative delivery system for OAg [10,39]. In GMMA, special physico-chemical properties of nano-sized particles are combined with the presentation of multiple saccharide epitopes. GMMA are self-adjuvanting, naturally possessing Toll-like receptor agonists and contain protein antigens that could contribute to the overall induced immune response. Traditional glycoconjugation is a complex multi-step process, comprising OAg extraction and purification, followed by OAg derivatization before conjugation to a carrier protein. In addition to the final conjugate, all intermediates need to be fully characterized. On the contrary, following fermentation of the GMMA-producing bacterial strains, two simple tangential flow filtration steps allow us to purify high yields of GMMA [10,40]. GMMA are complex systems, but a large panel of analytical methods have been developed to allow their full characterization [27,28]. Many of these methods can be applied to GMMA from different pathogens, as verified here for *S. flexneri* 6. The potential for strong immune responses and the simplicity of manufacture make the GMMA approach particularly attractive for global health vaccine production in LMIC, where the high cost of manufacture can be an obstacle to vaccine implementation [41]. 

Here, a wild-type *S. flexneri* serotype 6 strain has been successfully mutated for GMMA production. We have verified that *S. flexneri* 6 strains display OAg populations of different average size, including a very long G4C, similar to *S. sonnei* [42]. Interestingly, we have confirmed that the same OAg populations present on the bacterial surface are displayed with similar relative ratios in naturally released OMV and maintained in GMMA from mutated strains. Mutation of the GMMA producer strain to avoid G4C polymerization has confirmed that the HMM fraction detected by HPLC-SEC and NMR was actually a PS with the same structure of the OAg repeats but without the core region of LPS molecules.

Sugar length could have an impact on the immune response induced by GMMA as well as traditional glycoconjugates [39] and needs to be investigated for the design of an optimal vaccine against *S. flexneri* 6. For this reason, we have generated a panel of GMMA and glycoconjugates differing in sugar length. With both technologies, we have verified no major role of sugar length on the ability of the candidate vaccines to induce anti-OAg IgG antibodies with functional activity. Even very low OAg chain lengths, with an average of 1.5 repeating units, were able to induce functional IgG antibodies similar to longer OAg chains when presented both on GMMA or linked to a carrier protein. MMM and LMM OAg, but not HMM G4C, induced a pure T-dependent response when conjugated to CRM_197_, as verified in T-cell deficient mice. In contrast, all GMMA, independent of sugar length, induced a significant IgG response in T-cell deficient mice, even if the antibody levels were significantly lower than those induced in wild-type mice, highlighting the induction of a mixed T-dependent/T-independent response. Shortening the OAg chain length on GMMA led to a reduction of the T-independent component of the immune response induced. A pure T-dependent response could have a positive impact on memory and persistency of the antibody response. However, when compared head to head in a mouse study, we did not observe differences in the ability of GMMA or the glycoconjugate to induce strong responses after one only dose, re-injection, or in the persistence of the antibody response induced. 

Not only sugar length, but also sugar loading and conjugation chemistry are parameters that could impact the immune response induced by glycoconjugate vaccines. Here, we have verified that all these parameters do not have an impact on the induced immune response under the conditions tested. Also, OAg O-acetylation was verified not to be critical for the immune response induced by *S. flexneri* 6 OAg-based vaccines. All this information is important for the design of an optimal vaccine against *S. flexneri* 6.

In the absence of Alhydrogel, GMMA induced significantly higher anti-OAg specific IgG and stronger SBA titers than the conjugate. Different was the situation in the presence of Alhydrogel, where GMMA and conjugate induced similar levels of functional antibodies. In a previous study where we compared non-typhoidal *Salmonella* (NTS) GMMA and glycoconjugates [39], in the presence of Alhydrogel, GMMA induced anti-OAg IgG titers similar to glycoconjugates, but with much stronger functionality.

By looking at IgM and IgG subclasses, NTS glycoconjugates mainly induced IgG1, while increased Ig isotype-switching and induction of IgM was observed with GMMA. Differently, in this study, the *S. flexneri* 6 glycoconjugate not only induced IgG1, but also IgM, Ig2a, Ig2b, Ig3, at comparable levels with those induced by GMMA. The reason for this difference could be related to the different structure of *S. flexneri* 6 OAg compared to NTS OAg, or to a different contribution of the protein antigens to the overall immune response induced by NTS GMMA compared to *S. flexneri* 6 GMMA. Contribution of the protein antigens to the immune response induced by GMMA is currently under evaluation for GMMA from different pathogens.

## 5. Conclusions

Glycoconjugates are a well-established bacterial vaccine approach but can be costly, particularly when multi-component preparations are required, as in *Shigella*. Taken together, the results from this work indicate that with similar immunogenicity and a simpler manufacturing process, GMMA appear a promising strategy for the development of a vaccine against *Shigella flexneri* 6.

## Figures and Tables

**Table 1:** *Shigella flexneri* 6 bacterial strains used in this study.

Name in the Study	Phenotype	Country of Infection, Year of Isolation
Sf6_Sh10.3933	Wild type	Nigeria, 2010
Sf6_Sh10.6306	Wild type	India, 2010
Sf6_Sh10.6237	Wild type	Mexico, 2010
Sf6_Sh10.8537	Wild type	Egypt, 2010
H130920152	Wild type	Unknown
Sf6_Sh10.8537 ∆*tolR*	Hyper-blebbing	Produced in this study
Sf6_Sh10.8537 ∆*tolR* ∆*ept-etk*	Hyper-blebbing, no G4C produced	Produced in this study
Sf6_Sh10.8537 ∆*tolR* ∆*ept-etk* ∆*wzzB*	Hyper-blebbing, no G4C produced, short OAg produced	Produced in this study

**Table 2:** List of primers used in this study.

Primer Name	Sequence (5′ → 3′)
***tolR*** **KO F**	ACCGCCAGGCGTTTACCGTTAGCGAGAGCAACAAGGGGTAAGCCATGGCCGTGTAGGCTGGAGCTGCTTC
***tolR*** **KO R**	ACCCGCTCTCTTTCAAGCAAGGGAAACGCAGATGTTTAGATAGGCTGCGTCATATGAATATCCTCCTTAG
***ept-etk*** **KO F**	TTACTCTTTCTCGGAGTAACTATAACCGTAATAGTTATAGCCGTAACTGTGTCTTGAGCGATTGTGTAGG
***ept-etk*** **KO R**	AATATCTATCCCGTCACGCCAGGATTGATTGATCAGTTGCGCGCCAAACCTCCTCCTTAGTTCCTATTCC
***wzzB*** **KO F**	TCCCTTTGTAATAATTCATTATTTTTATCATTTATCCTATAGCATTCATGGTGTAGGCTGGAGCTGCTTC
***wzzB*** **KO R**	CGGGCAAGGTGTCACCACCCTGCCCCTTTTCTTTAAAACCGAAAAGATTACATATGAATATCCTCCTTAG

**Table 3:** All *Shigella flexneri* 6 wild-type strains analyzed display HMM G4C and MMM OAg of similar average size and relative amount compared to the corresponding OMV.

Strains	Average MM (KDa) and relative % * of HMM G4C and MMM OAg			
	Bacteria		OMV	
	HMM G4C	MMM OAg	HMM G4C	MMM OAg
Sf6_Sh10.3933	162.6 (46%)	17.8 (54%)	212.7 (52%)	16.5 (48%)
Sf6_Sh10.6306	165.0 (41%)	18.6 (59%)	197.4 (50%)	16.5 (50%)
Sf6_Sh10.6237	171.5 (42%)	18.0 (58%)	208.9 (42%)	16.4 (58%)
Sf6_Sh10.8537	151.3 (28%)	16.7 (72%)	205.2 (43%)	16.7 (57%)
H130920152	162.5 (59%)	17.1 (41%)	220.4 (64%)	16.4 (36%)

* Calculated as ratio % of the peak areas detected in HPLC-SEC dRI chromatograms.

**Table 4:** Main characteristics of conjugates obtained with sugars of different lengths and by different chemistry.

Chemistry	Conjugates	OAg Average Size (KDa)	% OAg O-Acetylation	OAg/Protein w/w Ratio (Molar Ratio)	% Free OAg
Random	G4C-deOAc-ox-CRM_197_	174	0	0.29	9
	G4C-ox-CRM_197_	174	63	0.38	14
	MMMOAg-ox-CRM_197_	22	66	0.54	<5
Selective	MMMOAg-ADH-SIDEA- CRM_197_	22	66	1.58 (4.2)	<5
	LMMOAg-ADH-SIDEA-CRM_197_	1.7	52	0.085 (4.9)	<10

**Table 5:** Main characteristics of GMMA differing in sugar length.

GMMA	nmol Lipid A/mg GMMA Protein	Total Sugar/Protein w/w Ratio	Z Average Diameter nm (PdI)	2 x Rw nm
∆*tolR*	134.7	0.47	110.5 (0.1)	83.2
∆*tolR* ∆G4C	177.4	0.53	103.2 (0.1)	82.2
∆*tolR* ∆G4C ∆*wzzB*	187.6	0.11	83.0 (0.1)	72.8

## Supplementary Materials

The following are available online at https://www.mdpi.com/2076-393X/8/2/160/s1, Figure S1: ^1^H and monodimensional diffusion-ordered spectroscopy (DOSY) for measuring diffusion data of polysaccharide purified from Sf6_Sh10.8537, optimizing the gradient pulse length, diffusion time, and the intensity of z-gradient; Figure S2: ^1^H NMR of de-O-acetylated G4C (bottom spectrum) compared to O-acetylated native one (upper spectrum); Figure S3: *S. flexneri* 6 glycoconjugates (A) and GMMA (B) differing for sugar length compared in mice. Eight CD1 mice per group were s.c. immunized at days 0 and 28, with 1 µg OAg dose on Alhydrogel (glycoconjugates) or 0.5 µg OAg dose without Alhydrogel (GMMA). SBA titers of single sera collected at day 42 from each group against *S. flexneri* 6 strains.
