# Supplementary material for: GMMA and Glycoconjugate Approaches Compared in Mice for the Development of a Vaccine against Shigella flexneri Serotype 6
Source: Vaccines (Basel). 2020 Apr 3;8(2):160. doi: 10.3390/vaccines8020160 (PMC7349896; doi:10.3390/vaccines8020160)
Supplement: Supplementary file 1 [file vaccines-08-00160-s001.pdf]

vaccines

GMMA and Glycoconjugate Approaches Compared in Mice for the Development of a Vaccine against *Shigella flexneri* Serotype 6

Maria Michelina Raso [1,2], Gianmarco Gasperini [1], Renzo Alfini [1], Fabiola Schiavo [1], Maria Grazia Aruta [1], Martina Carducci [1], Maria Concetta Forgione [3], Silvia Martini [3], Paola Cescutti [2], Francesca Necchi [1] and Francesca Micoli [1,*]

1 GSK Vaccines Institute for Global Health (GVGH) S.r.l., via Fiorentina 1, 53100 Siena, Italy; maria-michelina.m.raso@gsk.com (M.M.R.); gianmarco.x.gasperini@gsk.com (G.G.); renzo.x.alfini@gsk.com (R.A.); fabiola.schiavo@yahoo.it (F.S.); maria-grazia.x.aruta@gsk.com (M.G.A.); martina.x.carducci@gsk.com (M.C.); francesca.x.necchi@gsk.com (F.N.); francesca.x.micoli@gsk.com (F.M.)

2 Department of Life Science, University of Trieste, Building C11, via L. Giorgieri 1, 34127 Trieste, Italy; mariamichelina.raso@phd.units.it (M.M.R.); pcescutti@units.it (P.C.)

3 GSK, via Fiorentina 1, 53100 Siena, Italy; mariaconcetta.x.forgione@gsk.com (M.C.F.); silvia.x.martini@gsk.com (S.M.)

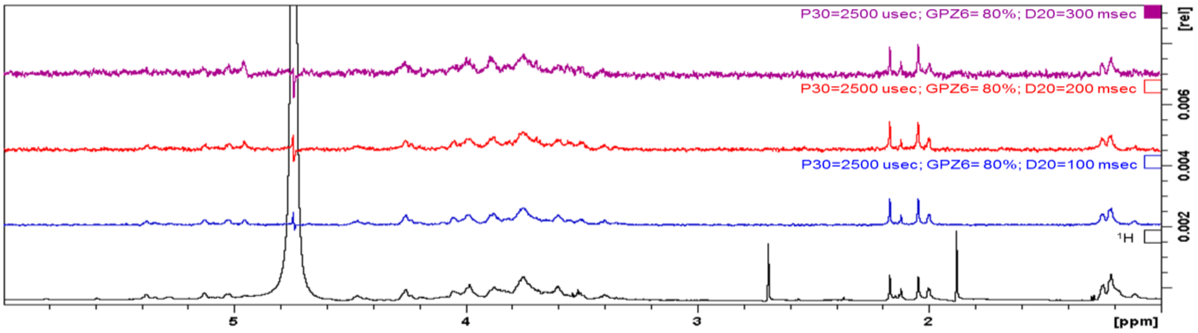

Figure S1. ^{1}H and monodimensional Diffusion-ordered spectroscopy (DOSY) for measuring diffusion data of polysaccharide purified from Sf6_Sh10.8537 optimizing the gradient pulse length, diffusion time and the intensity of z-gradient.

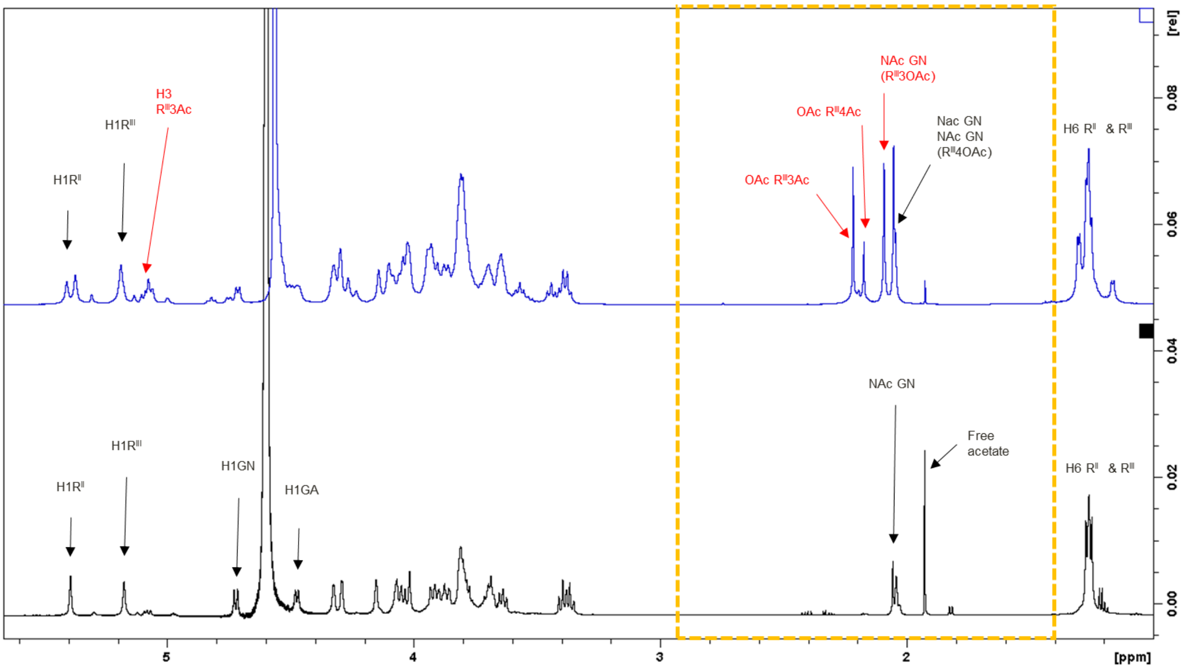

Figure S2. ^{1}H NMR of de-O-acetylated G4C (bottom spectrum) compared to O-acetylated native one (upper spectrum).

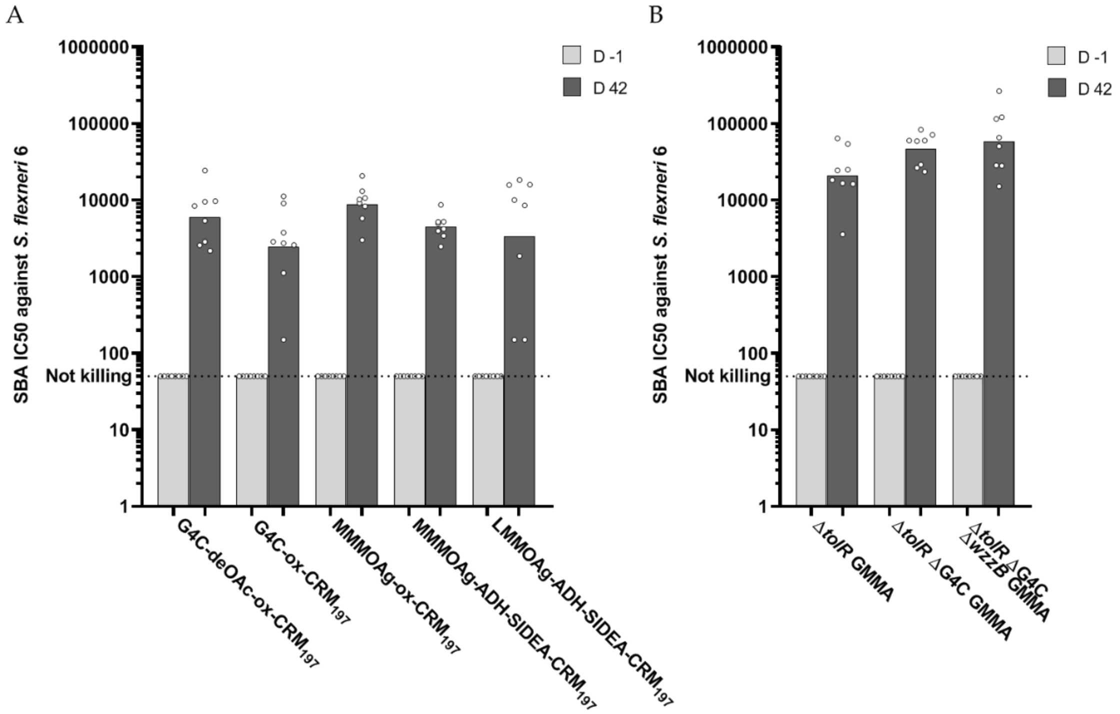

Figure S3. *S. flexneri* 6 glycoconjugates (A) and GMMA (B) differing for sugar length compared in mice. Eight CD1 mice per group were s.c. immunized at days 0 and 28, with 1 μg OAg dose on Alhydrogel (glycoconjugates) or 0.5 μg OAg dose without Alhydrogel (GMMA). SBA titers of single sera collected at day 42 from each group against *S. flexneri* 6 strain.
